# Checklist of the family Chloropidae (Diptera) of Finland

**DOI:** 10.3897/zookeys.441.7505

**Published:** 2014-09-19

**Authors:** Emilia Nartshuk, Jere Kahanpää

**Affiliations:** 1 Zoological Institute of the Russian Academy of Sciences, Universitetskaya nab. 1 St.-Petersburg 199034 Russia; 2 Finnish Museum of Natural History, Zoology Unit, P.O. Box 17, FI-00014 University of Helsinki, Finland

**Keywords:** Finland, Chloropidae, species list, biodiversity, faunistics

## Abstract

A checklist of 147 species the Chloropidae (Diptera) recorded from Finland. *Centorisoma elegantulum* Becker is recorded for the first time from Finland.

## Introduction

The Chloropidae is a large family of acalyptrate Diptera. It belongs to superfamily Carnoidea with Milichiidae as the closest relative. The classification of the family used follows Andersson (1977) and Nartshuk (1983).

The North European chloropid fauna has recently been revised by Nartshuk and Andersson (2013). Details of Finnish chloropid literature, species distribution and ecology, and other details can be found in their book. In comparison with the neighbouring countries, Finland ranks second in the number of chloropid species after Sweden (189 species) but well ahead of Denmark (119 species) and Norway (97 species). The genera *Gaurax*, *Incertella* and *Rhopalopterum* need more attention as there are several species in these genera known from neighbouring countries but not from Finland.

This checklist is based mostly on museum material in the Finnish Natural History museum (MZH) studied by E. Nartshuk. The Finnish species were last listed in Nartshuk and Andersson (2013).

**Number of species:**

World: 2880 species (Pape et al. 2011)

Europe: 394 species

Finland: 149–150 species

Faunistic knowledge level in Finland: average

## Checklist

suborder Brachycera Macquart, 1834

clade Eremoneura Lameere, 1906

clade Cyclorrhapha Brauer, 1863

infraorder Schizophora Becher, 1882

clade Muscaria Enderlein, 1936

parvorder Acalyptratae Macquart, 1835

superfamily Carnoidea Newman, 1834

**CHLOROPIDAE** Rondani, 1856

CHLOROPINAE Rondani, 1856

***CENTORISOMA*** Hendel, 1907

*Centorisoma elegantulum* Becker, 1910 (see Notes)

***CETEMA*** Hendel, 1907

= ***Centor*** Loew, 1866 preocc.

**sg. *Cetema*** Hendel, 1907

*Cetema cereris* (Fallén, 1820)

*Cetema elongatum* (Meigen, 1830)

*Cetema myopinum* (Loew, 1866)

*Cetema neglectum* Tonnoir, 1921

*Cetema simile* Ismay, 1985

***CHLOROPS*** Meigen, 1803

= ***Oscinis*** Latreille, 1804

**sg. *Chlorops*** Meigen, 1803

*Chlorops anthracophagoides* Strobl, 1900 (see Notes)

*Chlorops calceatus* Meigen, 1830

*Chlorops centromaculatus* Duda, 1933

*Chlorops geminatus* Meigen, 1830

*Chlorops gracilis* Meigen, 1830

*Chlorops hypostigma* Meigen, 1830

*Chlorops kirigaminensis* Kanmiya, 1978

= *zonulatus* auct. nec Wahlgren, 1913

*Chlorops laetus* Meigen, 1830

*Chlorops meigenii* Loew, 1866

= *rufescens* Oldenberg, 1923

*Chlorops nigripalpis* (Duda, 1933)

= *crassipalpis* Smirnov, 1958

*Chlorops obscurellus* (Zetterstedt, 1838)

= *brunnipes* auct. nec (Zetterstedt, 1848)

*Chlorops palpatus* Smirnov, 1959

*Chlorops pannonicus* Strobl, 1893

*Chlorops planifrons* (Loew, 1866)

= *triangularis* Becker, 1910

*Chlorops pumilionis* (Bjerkander, 1778)

*Chlorops ringens* Loew, 1866

*Chlorops riparius* Smirnov, 1958

*Chlorops rossicus* Smirnov, 1955

*Chlorops rufinus* (Zetterstedt, 1848)

= *citrinellus* (Zetterstedt, 1848)

*Chlorops scalaris* Meigen, 1830

*Chlorops scutellaris* (Zetterstedt, 1838)

= *laevicollis* Becker, 1910

= *freyi* Duda, 1933

*Chlorops serenus* Loew, 1866

*Chlorops speciosus* Meigen, 1830

*Chlorops troglodytes* (Zetterstedt, 1848)

**sg. *Sclerophallus*** Beschovski, 1978

*Chlorops limbatus* Meigen, 1830

= *brevimanus* Loew, 1866

*Chlorops varsoviensis* Becker, 1910

***CHLOROPSINA*** Becker, 1911

*Chloropsina distinguenda* (Frey, 1909)

***CRYPTONEVRA*** Lioy, 1864

*Cryptonevra diadema* (Meigen, 1830)

*Cryptonevra flavitarsis* (Meigen, 1830)

***DIPLOTOXA*** Loew, 1863

*Diplotoxa messoria* (Fallén, 1820)

***DIPLOTOXOIDES*** Andersson, 1977

*Diplotoxoides dalmatina* (Strobl, 1900)

***EPICHLOROPS*** Becker, 1910

*Epichlorops puncticollis* (Zetterstedt, 1848)

***EUTROPHA*** Loew, 1866

*Eutropha fulvifrons* (Haliday, 1833)

*Eutropha variegata* Loew, 1866

***LASIOSINA*** Becker, 1910

*Lasiosina albipila* (Loew, 1866)

*Lasiosina brevisurstylata* Dely-Draskovits, 1977

*Lasiosina herpini* (Guérin-Méneville, 1843)

= *cinctipes* auct. nec (Meigen, 1830)

*Lasiosina parvipennis* Duda, 1933

***MELANUM*** Becker, 1910

*Melanum laterale* (Haliday, 1833)

***MEROMYZA*** Meigen, 1830

**sg. *Meromyza*** Meigen, 1830

*Meromyza curvinervis* (Zetterstedt, 1848)

= *hybrida* Péterfi, 1961

*Meromyza elbergi* Fedoseeva, 1979

*Meromyza ingrica* Nartshuk, 1992

*Meromyza mosquensis* Fedoseeva, 1960

*Meromyza nigriseta* Fedoseeva, 1960

*Meromyza nigriventris* Macquart, 1835

= *rostrata* Hubicka, 1966

*Meromyza ornata* (Wiedemann, 1817)

= *sororcula* Fedoseeva, 1962

*Meromyza palposa* Fedoseeva, 1960

*Meromyza pluriseta* Péterfi, 1961

*Meromyza pratorum* Meigen, 1830

*Meromyza rohdendorfi* Fedoseeva, 1974

*Meromyza saltatrix* (Linnaeus, 1761)

*Meromyza sibirica* Fedoseeva, 1961

*Meromyza triangulina* Fedoseeva, 1960

*Meromyza variegata* Meigen, 1830

= *lidiae* Nartshuk, 1992

*Meromyza zimzerla* Nartshuk, 1992

***NEOHAPLEGIS*** Beschovski, 1981

*Neohaplegis tarsata* (Fallén, 1820)

***PLATYCEPHALA*** Fallén, 1820

*Platycephala planifrons* (Fabricius, 1798)

*Platycephala umbraculata* (Fabricius, 1794)

***PSEUDOPACHYCHAETA*** Strobl, 1902

*Pseudopachychaeta approximatonervis* (Zetterstedt, 1848)

*Pseudopachychaeta oscinina* (Fallén, 1813)

= *heleocharis* (Nartshuk, 1964)

*Pseudopachychaeta ruficeps* (Zetterstedt, 1838)

***THAUMATOMYIA*** Zenker, 1833

*Thaumatomyia glabra* (Meigen, 1830)

*Thaumatomyia hallandica* Andersson, 1966

*Thaumatomyia notata* (Meigen, 1830)

*Thaumatomyia rufa* (Macquart, 1835)

*Thaumatomyia trifasciata* (Zetterstedt, 1848)

***TRICHIEURINA*** Duda, 1933

*Trichieurina pubescens* (Meigen, 1830)

OSCINELLINAE Becker, 1910

***APHANOTRIGONUM*** Duda, 1932

*Aphanotrigonum cinctellum* (Zetterstedt, 1848)

= *fasciella* (Zetterstedt, 1855)

*Aphanotrigonum hungaricum* Dely-Draskovits, 1981

*Aphanotrigonum nigripes* (Zetterstedt, 1848)

*Aphanotrigonum trilineatum* (Meigen, 1830)

= *beschovskii* Dely-Draskovits, 1981

***ASPISTYLA*** Duda, 1933

*Aspistyla plumiger* (Meigen, 1830)

***CALAMONCOSIS*** Enderlein, 1911

**sg. *Calamoncosis*** Enderlein, 1911

*Calamoncosis aprica* (Meigen, 1830)

*Calamoncosis duinensis* (Strobl, 1909)

*Calamoncosis minima* (Strobl, 1893)

*Calamoncosis oscinella* (Becker, 1910)

**sg. *Rhaphiopyga*** Nartshuk, 1971

*Calamoncosis glyceriae* Nartshuk, 1958

***COLLINIELLA*** Nartshuk & Andersson, 2013

*Colliniella meijerei* (Duda, 1932) (see Notes)

***CONIOSCINELLA*** Duda, 1929

*Conioscinella frontella* (Fallén, 1820)

*Conioscinella gallarum* (Duda, 1933)

*Conioscinella livida* Nartshuk, 1970

*Conioscinella mimula* Collin, 1946

*Conioscinella sordidella* (Zetterstedt, 1848)

*Conioscinella zetterstedti* Andersson, 1966

***DICRAEUS*** Loew, 1873

**sg. *Dicraeus*** Loew, 1873

*Dicraeus tibialis* (Macquart, 1835)

**sg. *Oedesiella*** Becker, 1910

*Dicraeus fennicus* Duda, 1932

*Dicraeus rossicus* Stackelberg, 1955

**sg. *Paroedesiella*** Anonymous in Imperial Institute of Entomology, 1937

*Dicraeus nitidus* Wahlgren, 1913

= *napaeus* Collin, 1946

*Dicraeus styriacus* (Strobl, 1898)

= *vallaris* Collin, 1946

*Dicraeus vagans* (Meigen, 1838)

***ELACHIPTERA*** Macquart, 1835

*Elachiptera cornuta* (Fallén, 1820)

*Elachiptera diastema* Collin, 1946

*Elachiptera scrobiculata* (Strobl, 1901)

= *tenuiseta* (Frey, 1947)

*Elachiptera tuberculifera* (Corti, 1909)

***ERIBOLUS*** Becker, 1910

*Eribolus gracilior* (de Meijere, 1918)

*Eribolus hungaricus* Becker, 1910

*Eribolus nana* (Zetterstedt, 1838)

= *sudeticus* Becker, 1910

*Eribolus slesvicensis* Becker, 1910

***GAMPSOCERA*** Schiner, 1862

*Gampsocera numerata* (Heeger, 1858)

***GAURAX*** Loew, 1863

*Gaurax borealis* (Duda, 1933)

*Gaurax dubius* (Macquart, 1835)

*Gaurax ephippium* (Zetterstedt, 1848)

= *strobilum* Karps, 1981

*Gaurax fascipes* Becker, 1910

*Gaurax macrocerus* (Nartshuk, 1962)

*Gaurax maculipennis* (Zetterstedt, 1848)

***HAPLEGINELLA*** Duda, 1933

*Hapleginella laevifrons* (Loew, 1858)

***INCERTELLA*** Sabrosky, 1980

*Incertella albipalpis* (Meigen, 1830)

*Incertella kerteszi* (Becker, 1910)

*Incertella nigrifrons* (Duda, 1933)

*Incertella scotica* (Collin, 1946)

*Incertella zuercheri* (Duda, 1933)

***LASIAMBIA*** Anonymous in Imperial Instutute of Entomology, 1937

= *Lasiambia* Enderlein, 1936

= *Lasiambia* Sabrosky, 1941

*Lasiambia brevibucca* (Duda, 1932)

*Lasiambia coxalis* (von Roser, 1840)

= *oophila* (Hennig, 1941)

*Lasiambia palposa* (Fallén, 1820)

***LIPARA*** Meigen, 1830

*Lipara lucens* Meigen, 1830

*Lipara pullitarsis* Doskočil & Chvála, 1971

= *rufitarsis* misid.

***MICROCERCIS*** Beschovski, 1978

*Microcercis trigonella* (Duda, 1933)

***OSCINELLA*** Becker, 1909

**sg. *Oscinella*** Becker, 1909

*Oscinella angularis* Collin, 1946

*Oscinella cariciphila* Collin, 1946

*Oscinella frit* (Linnaeus, 1758)

*Oscinella maura* (Fallén, 1820)

= *albiseta* (Meigen, 1830)

*Oscinella nigerrima* (Macquart, 1835)

*Oscinella nitidissima* (Meigen, 1838)

*Oscinella pusilla* (Meigen, 1830)

*Oscinella ventricosi* Nartshuk, 1956

*Oscinella vindicata* (Meigen, 1830)

= *hortensis* Collin, 1946

***OSCINIMORPHA*** Lioy, 1864

*Oscinimorpha minutissima* (Strobl, 1900)

*Oscinimorpha sordidissima* (Strobl, 1893)

***OSCINISOMA*** Lioy, 1864

*Oscinisoma cognatum* (Meigen, 1830)

***POLYODASPIS*** Duda, 1933

*Polyodaspis ruficornis* (Macquart, 1835)

*Polyodaspis sulcicollis* (Meigen, 1838)

***RHOPALOPTERUM*** Duda, 1929

*Rhopalopterum anthracinum* (Meigen, 1930)

*Rhopalopterum atricillum* (Zetterstedt, 1838)

= *atripes* (Duda, 1933)

*Rhopalopterum atricorne* (Zetterstedt, 1838)

= *platythorax* (Nartshuk, 1958)

*Rhopalopterum brunneipenne* Beschovski & Lansbury, 1987

*Rhopalopterum fasciolum* (Meigen, 1830)

*Rhopalopterum femorale* (Collin, 1946)

***SIPHONELLA*** Macquart, 1835

*Siphonella oscinina* (Fallén, 1820)

***SIPHUNCULINA*** Rondani, 1856

*Siphunculina aenea* (Macquart, 1835)

***SPECCAFRONS*** Sabrosky, 1980

*Speccafrons halophila* (Duda, 1933)

***TRACHYSIPHONELLA*** Enderlein, 1936

*Trachysiphonella ruficeps* (Macquart, 1835)

= *pygmaea* (Meigen, 1838)

*Trachysiphonella scutellata* (von Roser, 1840)

***TRICIMBA*** Lioy, 1864

**sg. *Nartshukiella*** Beschovski, 1981

*Tricimba cincta* (Meigen, 1830)

= *sulcella* (Zetterstedt, 1848)

**sg. *Tricimba*** Lioy, 1864

*Tricimba lineella* (Fallén, 1820)

## Excluded species

*Chlorops figuratus* (Zetterstedt, 1848) not found within present borders.

## Notes

***Centorisoma elegantulum* Becker, 1910**. First records from Finland: 1 ex Ta: Heinola, Lusi, July 2013, leg. J. Kahanpää; 6 exx N: Loviisa, 2013, leg. Jari Flinck.

***Chlorops anthracophagoides* Strobl, 1900**. Recorded in Nartshuk (1998) from Eriksberg, which is a Finnish locality.

***Colliniella meijerei* (Duda, 1932)**. Recently added to the list of Finnish Diptera (Kahanpää 2013).
